# Tumor Suppressor MicroRNAs in Clinical and Preclinical Trials for Neurological Disorders

**DOI:** 10.3390/ph17040426

**Published:** 2024-03-27

**Authors:** Austin Lui, Timothy Do, Omar Alzayat, Nina Yu, Su Phyu, Hillary Joy Santuya, Benjamin Liang, Vidur Kailash, Dewey Liu, Sabra S. Inslicht, Kiarash Shahlaie, DaZhi Liu

**Affiliations:** 1 Department of Neurology, University of California at Davis, Davis, CA 95616, USA; alui@ucdavis.edu (A.L.); vkailash2@student.touro.edu (V.K.);; 2 Department of Neurological Surgery, University of California at San Francisco, San Francisco, CA 94143, USA; 3 Department of Psychiatry and Behavioral Sciences, University of California at San Francisco, San Francisco, CA 94143, USA; 4 San Francisco VA Health Care System, San Francisco, CA 94121, USA; 5 Department of Neurological Surgery, University of California at Davis, Davis, CA 95616, USA; 6 Mirnova Therapeutics Inc., Davis, CA 95618, USA

**Keywords:** tumor suppressor microRNA, cancers, neurological disorders

## Abstract

Cancers and neurological disorders are two major types of diseases in humans. We developed the concept called the “Aberrant Cell Cycle Disease (ACCD)” due to the accumulating evidence that shows that two different diseases share the common mechanism of aberrant cell cycle re-entry. The aberrant cell cycle re-entry is manifested as kinase/oncoprotein activation and tumor suppressor (TS) inactivation, which are associated with both tumor growth in cancers and neuronal death in neurological disorders. Therefore, some cancer therapies (e.g., kinase/oncogene inhibition and TS elevation) can be leveraged for neurological treatments. MicroRNA (miR/miRNA) provides a new style of drug-target binding. For example, a single tumor suppressor miRNA (TS-miR/miRNA) can bind to and decrease tens of target kinases/oncogenes, producing much more robust efficacy to block cell cycle re-entry than inhibiting a single kinase/oncogene. In this review, we summarize the miRNAs that are altered in both cancers and neurological disorders, with an emphasis on miRNA drugs that have entered into clinical trials for neurological treatment.

## 1. Introduction

**Figure 1:**
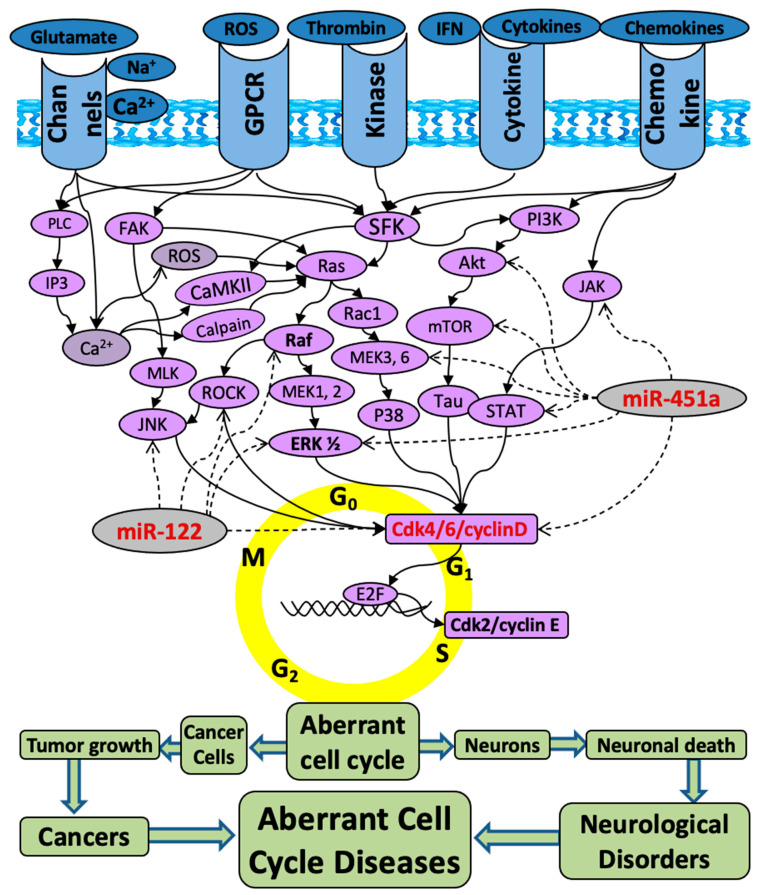
The schematic of Aberrant Cell Cycle Diseases (ACCDs). The molecules and related mitogenic pathways contributing to the aberrant cell cycle re-entry that is associated with not only tumorigenesis in cancers but also neuronal death in neurological diseases. The solid arrow lines indicate activation, while the dashed arrow lines indicate inhibition. The arrows do not necessarily indicate direct binding and/or activation of the downstream effectors; intermediate molecules may exist. Akt: protein kinase B; Ca^2+^: calcium; Cdk: cyclin-dependent kinase; ERK: extracellular signal-regulated kinase; FAK: focal adhesion kinase; GPCR: G protein-coupled receptor; GSK3β: glycogen synthase-3 beta; IP3: inositol trisphosphate; JAK: Janus kinase; JNK: c-Jun N-terminal kinases; MEK: mitogen-activated protein kinase kinase; miR-122: microRNA-122; miR-451a: microRNA-451a; MLK: mixed lineage kinases; mTOR: mammalian target of rapamycin; NF-kB: nuclear factor kappa B; PI3K: phosphatidylinositol 3-kinase; PLC: phospholipase C; Ras: rat sarcoma virus kinase; Rac1: ras-related C3 botulinum toxin substrate 1; Raf: rapidly accelerated fibrosarcoma; ROS: reactive oxygen species; SFKs: Src family kinases; STAT: signal transducer and activator of transcription.

We developed a novel concept of “Aberrant Cell Cycle Disease” (ACCD), revealing that two major types of diseases (cancers and neurological diseases) share the same mechanism of “aberrant cell cycle re-entry”. The aberrant cell cycle is manifested as oncogenic kinase activation and/or tumor suppressor inhibition, which are hallmarks of both tumor growth in cancers and neuronal death in neurological disorders [1]. Based on the ACCD concept, the key cell cycle players can be expanded from cyclin and cyclin-dependent kinases (CDKs) to Src family kinase (SFK), Jun N-terminal kinase (JNK), extracellular signal-regulated kinase (ERK), and other many oncogenic kinases (Figure 1) [1].

It is well known that genetic, environmental, and other factors cause cancers, which is featured as the aberrant cell cycle (unlimited cell proliferation) of cancer cells [1]. As for mature neurons, they are post-mitotic cells and unable to pass the mitotic phase to regain the stable, resting G0 phase of the cell cycle. Considering tat the cell cycle is an irreversible process, mature neurons ultimately die once they re-enter the cell cycle aberrantly under certain conditions [1]. Therefore, we concluded that the aberrant cell cycle is a common mechanism of both cancers and neurological disorders [1], as blocking the cell cycle can kill cancer cells for cancer therapy and rescue mature neurons for neurological therapy.

There are in general two strategies to block the cell cycle, namely the inhibition of oncogenic factors (e.g., kinases) and the elevation of tumor suppressors. Focusing on the oncogenic kinases, we recently reviewed all 74 FDA-approved kinase inhibitors (mostly for cancer therapy) by the end of 2022 in preclinical and clinical trials for neurological treatment, with nearly 500 references supporting the possibility of repurposing kinase inhibitors for the treatment of neurological disorders, such as Alzheimer’s disease (AD), intracerebral hemorrhage (ICH), ischemic stroke (IS), traumatic brain injury (TBI), and other disorders [2].

However, most current drug-development efforts (including kinase inhibitors) are based on the “receptor theory”—the basic framework for drug development for more than a century—which focuses on small molecules, with each molecule targeting a single gene, protein, or enzyme. The drugs developed by the traditional “one-drug-on-one-target” binding style may not be effective for the treatment of neurological disorders in humans, because (1) neurological disorders are heterogeneous in general with different causes, severities, and genetic backgrounds of patients and (2) increasing evidence shows that a large number of oncogenic kinases are significantly increased in neurological disorders [2]. Indeed, the selected testing of hundreds of traditional compounds that are effective in animal models has led to little success in clinical trials for the treatment of neurological disorders in humans [3]. The combinatorial therapy “multi-drug-on-multi-target” may be effective, but determining the optimum combination and dynamic interaction has been a challenge.

MicroRNAs (miR/miRNA), a type of small non-coding RNA, are regarded as next-generation promising drug targets for various diseases, including cancers and neurological disorders [4,5,6,7,8]. This is mostly because miRNAs provide a new “one-drug-on-numerous-target” binding style—a single miRNA binds to the 3′ untranslated region (3′UTR) of hundreds of target genes [9,10], leading to post-translational suppression of those target genes [11,12,13,14,15]. For a tumor suppressor miRNA (TS-miR/TS-miRNA), it is capable of inhibiting tens of oncogenic kinases among hundreds of target genes (Figure 1). Therefore, elevating a TS-miR has the potential to inhibit numerous oncogenic kinases and may lead to much more robust efficacy compared to targeting a single kinase by a kinase inhibitor.

In this review, we summarize miRNAs that are altered in cancers and neurological disorders and highlight the TS-miRs that have been tested in experimental models and/or clinical trials for the treatment of neurological disorders to discuss the feasibility and applicability of leveraging TS-miRs for neurological treatment.

## 2. MicroRNA History and Biogenesis

MicroRNAs (miR/miRNA) are small non-coding RNAs, about ~22 nucleotides in length, that are capable of modulating gene expression. In 1993, the Ambros and Ruvkun groups reported that lin-4 gene coded a small 22-nucleotide RNA instead of a conventional protein-coding gene in their studies of the lin-4 gene in Caenorhabditis elegans [16,17,18,19,20,21]. Since lin-4 had a complementary sequence to the 3′ untranslated region (3’UTR) of another gene lin-14, and lin-14 was post-transcriptionally downregulated through binding the 3′ UTR of Lin-14 [16], they postulated that lin-4 could regulate lin-14 by a post-transcriptional mechanism [17]. This discovery revealed the existence of miRNAs while having limited significance outside of the *C. elegans* research community, until the discovery of the second miRNA, let-7, in 2000 [22]. Unlike lin-4, let-7 is conserved across many organisms, including humans, suggesting that these miRNAs should have a more general role in biology and the regulation of gene expression [23]. In addition to the discovery of the post-transcriptional miRNA-induced gene modulation, these findings unfolded a more complete picture of the components of the RNA-induced silencing complex (RISC) by which miRNA regulates gene expression, kicking off the new field of miRNA research [24,25,26,27,28,29,30].

**Figure 2:**
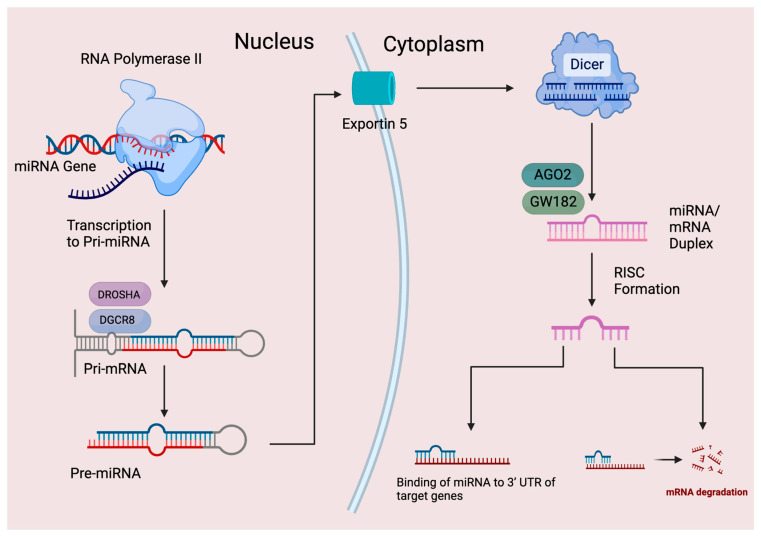
Biogenesis, processing, and function of miRNAs (AGO2: Argonaute 2 protein; GW182: glycine-tryptophan protein of 182 kDa; RISC: RNA-induced silencing complex; 3′UTR: 3′ untranslated region).

As shown in Figure 2, the miRNA biogenesis starts with transcribing a gene into a primary miRNA (pri-miRNA), which is 5′-capped and 3′-polyadenylated, with the transcription being performed by RNA Polymerase II. The pri-miRNAs are then cleaved by a microprocessor complex, consisting of RNA-binding protein DGCR8 and type III RNase Drosha, into a ~85-nucleotide stem–loop structure known as precursor miRNA (pre-miRNA) [31]. Pre-miRNA is exported and transported by Exportin 5 (XPO5) from the nucleus to the cytoplasm, where the pre-miRNA is processed by another RNase III enzyme Dicer into a mature miRNA. This creates the miRNA-induced silencing complex (miRISC), which consists of glycine-tryptophan protein (GW182) and Argonaute 2 protein, forming a complex of miRNA and mRNA interaction. MiRNAs usually interact with the 3′ UTR of their target genes post-transcriptionally to suppress expression under certain conditions, one of the conditions being RISC, but interaction with other regions of the gene, including the 5′ UTR, coding sequence, and even gene promoters has also been reported [32,33]. In addition, miRNAs are able to activate gene expression or control the rate of translation/transcription of genes [34,35]. miRNAs have even been shown to be in extracellular fluids where they may potentially serve as biomarkers or mediate cell-to-cell communication [36,37,38]. The aberrant expression of miRNAs is associated with many human diseases, showing that miRNAs are critical for normal human development and a variety of biological processes, making them a promising target for medical therapeutics [24,39,40].

## 3. MicroRNA Biomarkers in Diseases

In this section, we summarize miRNA fingerprints in different types of samples for each sub-type of disease. As shown in the Supplementary Table S1, there were (1) a total of 1132 miRNAs that were found to be dysregulated (upregulated or downregulated) across 15 types of cancers (sarcomas, gastrointestinal cancers, nervous system cancers, skin cancers, hepatobiliary cancers, bone cancers, urological cancers, breast cancers, lung cancers, female genital neoplasms, male genital neoplasms, heart cancers, head and neck cancers, eye cancers, adpancreatic cancers) in different types of samples, including plasma, serum, blood, exosomes, tissues, and cell lines, and (2) a total of 779 miRNAs were found to be dysregulated (upregulated or downregulated) across 7 types of neurological disorders (AD, PD, ALS, HD, Stroke, TBI, and seizures) in different sample types, including CSF, serum, plasma, blood, central nervous system tissue, exosomes, cell lines, and immune cells (Supplementary Materials).

### 3.1. MicroRNA Dysregulation and Biomarkers in Cancers

The discovery of emerging biomarkers is critical for cancers that require early identification and therapy. miRNAs can be detected in tissue and extracellular samples not limited to feces and blood [41] and are reported to be relatively stable in a variety of biological fluids as well as formalin-fixed paraffin-embedded tissues [42,43]. Extracellular miRNAs have been widely reported as potential biomarkers for various malignancies [44], with dysregulated circulating miRNAs being reported particularly in the early stages of various cancers [45].

Abnormal miRNA expression (i.e., upregulation or downregulation) in malignant cells is often due to changes in genomic miRNA copy numbers and gene locations pertaining to amplification, deletion or translocation, impacting multiple pathological processes in a variety of cancers. The earliest discovery of miRNA gene location change is the loss of miR-15a/16-1 cluster gene at chromosome 13q14, which is chronically seen in B-cell chronic lymphocytic leukemia patients [46]. In lung cancer, the 5q33 region containing miR-143 and miR-145 can often be deleted, leading to the downregulation of the respective miRNAs in question. Additionally, the amplification of the miR-17–92 cluster gene was observed in both lung cancer and B-cell lymphoma, and the translocation of the cluster gene was also observed in T-cell acute lymphoblastic leukemia, leading to the overexpression of the respective miRNAs [47].

It was reported that miR-29c was significantly downregulated in the plasma of hepatocellular carcinoma (HCC) patients [48], and abnormal miRNA expression is implicated in the carcinogenesis pathway, including the decrease in the apoptosis of malignant cells and increased proliferation [49,50]. It was also reported that the downregulation of miR-29a-3p is implicated in tumorigenesis and the development of colorectal cancer (CRC) and other cancers by increasing cell proliferation, migration, and invasion [51,52]. In prostate cancer (PCa), mouse models have revealed that the loss of miR-15 and miR-16 and the simultaneous overexpression of miR-21 promote cancer progression [53]. By decreasing levels of Bcl2, Mcl1, CCND1, and WNT3A, miR-15a and miR-16 exert tumor suppressor functions [54]. The administration in vivo of antagonistic miRNA directed against miR-15a and miR-16 produces prostate hyperplasia and leads to the development of a tumor-prone phenotype [54]. Furthermore, tumor regression, growth arrest, and apoptosis induction have been observed in miR-15a- and miR-16-reconstituted LNCaP xenograft tumors [55]. Therefore, the abnormal miRNA expression levels can be used as prognostic and diagnostic biomarkers in certain types of cancers [56,57].

### 3.2. MicroRNA Dysregulation and Biomarkers in Acute Neurological Disorders:

The dysregulation of miRNA is implicated in the pathology of acute brain injuries. Acute brain injuries encompass several neurological disorders, including acute ischemic stroke (AIS), intracerebral hemorrhage (ICH), traumatic brain injury (TBI), seizures, and others. We and others have reported that miRNAs are significantly changed in the blood and/or brain at different timepoints in both humans and experimental animals after different types of acute brain injuries [58,59,60]. Our studies have demonstrated that TS-miRNAs (including miR-122, miR-125b, miR-140, and others) are decreased in the blood in both humans and/or rats after AIS, ICH, and TBI [58,61]. Aside from the patent of miR-125b and miR-122 for TBI therapy [62], we demonstrated that miR-122 mimic has a 6 h therapeutic window to improve multiple outcomes after AIS in rats [61,63]. Another particular miRNA, miR-124, was shown to be downregulated in human AIS patients within 24 h of AIS and paradoxically upregulated approximately 48 to 72 h after AIS [64]. Interestingly, miR-124 was shown to have both beneficial and detrimental effects in the context of ischemia [65]. One possible interpretation was that miR-124 may trigger anti-inflammatory responses and attenuate apoptosis [66] but at the same time exert detrimental effects on synaptic plasticity and axonal growth [65]. Separate studies reported that miR-98 was downregulated [67], while miR-210, miR-191, miR-15a, and miR-16-1 were upregulated after AIS in rodent models [68,69,70].

In a recent study examining miRNA levels in veterans with chronic mild TBI, there were changes in levels of 32 miRNAs in plasma and 45 miRNAs in extracellular vesicles compared to control patients [71]. For example, in extracellular vesicles, miR-21-5p, miR-30d-5p, and miR-423-3p levels were changed compared to control patients, and plasma levels of miR-103b-1-5p, miR-106a-5p, and miR-132-5p were changed compared to control patients [71]. In human blood miRNA profiling studies, one study showed the upregulation of miR-16, miR-92a, and miR-765 in patients with severe TBI [8], and another study showed the downregulation of miR-425–5p and miR-502 in mild TBI patients at an early time period [72]. miR-425–5p levels were also shown to be a strong predictor of outcomes 6 months after mild TBI [72]. miR-212-5p was downregulated from 6 through 72 h after TBI in mice [73]. It was reported that miR-124-3p was upregulated at 3 and 14 h post-TBI but downregulated at 24 h post-TBI in mice [74], while miR-873a-5p was upregulated in human brain tissue after TBI [75]. Functional studies have revealed that miR-124-3p targeted Rela, an inhibitory transcription factor of ApoE that promotes the breakdown of amyloid-β (Aβ) plaques; ultimately alleviated neurodegeneration; inhibited inflammation; and promoted neurite outgrowth after TBI in rodents [74,76].

In various epilepsy models, miR-128, miR-124, miR-155, and miR-23b-3p levels were downregulated, while miR-134, miR-126a, miR-137, miR-129-5p, miR-203, miR-199a-5p, miR-22, miR-132, miR-324-5p, miR-34a, and miR-184 were upregulated [59,77,78,79]. In human studies, it was reported that miR-124 was downregulated in patients with epilepsy [80], while miR181a and miR-146a were upregulated [80,81]. Functional studies showed that miR-124 inhibited NMDA receptors, decreased expression of CREB1, and decreased anti-epileptogenic activity [80,82]. miR-134 regulated dendrite spine density and altered epileptogenesis [83,84], while miR181a inhibited caspase-3 and was associated with seizure-induced neuronal apoptosis [81]. miR-128 suppressed the expression of different types of ion channels and signaling components of the ERK2 signaling pathway [78,85], and miR-155 modulated brain-derived neurotrophic factor (BDNF) levels [86].

### 3.3. MicroRNA Dysregulation and Biomarkers in Neurodegenerative Disorders

Neurodegenerative diseases occur secondarily to the loss of neurons in the central nervous system, and the incidence typically increases with age [87,88]. As life expectancy continues to increase, more and more elderly people suffer from neurodegenerative diseases [89], such as Alzheimer’s Disease (AD), Parkinson’s Disease (PD), Amyotrophic Lateral Sclerosis (ALS), Huntington’s Disease (HD), and others. Currently, there are limited treatment options for neurodegenerative disorders [90,91], despite some supportive therapies.

AD is the most common form of dementia in people over the age of 65, and the disease is characterized by progressive neuronal loss and inflammation, further impacting cognitive function. The AD pathology is due to the deposition of Aβ in the brain, which is a potent mitogenic factor resulting in abnormal cell cycle re-entry and death of neurons [92,93]. Since Aβ is derived from the cleavage of amyloid precursor protein (APP) by beta-site APP-cleaving enzyme 1 (BACE1) and the β-secretase complex [94], targeting APP has the potential to delay AD progress. A series of in vitro studies revealed that several miRNAs are capable of modulating APP expression, such as miR-106a and miR-520c [95]; miR-16 and miR-101 [96]; and miR-147, miR-655, miR-323-3p, miR-644, and miR-153 [97]. Moreover, multiple miRNAs can indirectly inhibit APP by downregulating BACE1 [98]. Two separate studies demonstrated that miR-124 was decreased in the brains of AD patients [99], and elevating miR-124 was able to downregulate BACE1 expression in experimental AD models [100]. The inverse relationship was also observed between BACE1 levels and several miRNAs (e.g., miR-29c, miR-298, miR-328, miR-195) in multiple experimental AD models [101,102]. Additionally, miRNA dysregulation has been found to play a role in neuroinflammation in AD. Specifically, miR-146a has been observed in AD studies to be a potential target to reduce astrocytic inflammation and stymie cognitive impairment associated with AD [103,104,105]. The proposed mechanisms by which miR-146a exerts these effects include the downregulation of tumor necrosis factor (TNF) receptor-associated factor 6 and NF-kB and the switching of microglial phenotypes to secrete lower levels of pro-inflammatory factors and increase phagocytic function [103,106]. Since these inflammatory cytokines are mitotic and associated with cell cycle re-entry, the anti-inflammatory approach has the potential to rescue neurons by blocking cell cycle re-entry. In preclinical investigations, miR-132 has shown promise as a potential therapeutic target for alleviating AD symptoms and progression [107,108]. Walgrave et al. recently reported miR-132 replacement in adult mice with AD-restored adult hippocampal neurogenesis and memory deficits [108], corroborating other studies that had similar findings [109,110]. Inhibitors for miR-181a, a TS-miRNA that is downregulated in cancers [111,112,113], were found to decrease Tau protein in the hippocampus of mice to rescue memory deficits from AD [114]. Inhibitors of miR-124, a TS-miRNA also found to be downregulated in cancers [115,116,117], also reduced Tau hyperphosphorylation and rescued learning and memory [118].

PD is the second most common neurodegenerative disorder, and its symptoms are often attributed to a loss of dopaminergic neurons of the substantia nigra. The two genes typically involved in PD include a-synuclein (SNCA) and leucine-rich repeat kinase2 (LRRK). Since higher expression of SNCA and the expression of the three mutant forms of SNCA contribute to the insoluble aggregates that comprise the main structure of Lewy Bodies [119], the downregulation of SNCA is a possible mechanism in treating PD. miR-7 and miR-153 have been shown to inhibit the expression of SNCA [120]. The dysregulation of LRRK2, which is highly expressed in the brain, including the hippocampus and striatum [121], is associated with the pathogenesis of PD. An experiment demonstrated that the transfection of miR-205 in neurons expressing a PD-related LRKK2 R1441G mutant had a role in preventing neurite outgrowth [122]. Other miRNA types that have been identified as altered in PD also include miR-29-c-3p, miR-124, mir-150, miR-626, and miR-4639-5p [123,124]. In PD, there is a progressive loss of dopamine (DA) and attenuated DA signaling, α-synuclein-filled Lewy bodies, and increased neuroinflammation [125]. While Levodopa, DA receptor agonists, and several inhibitors are mainstay medications for PD treatment, interesting miRNA studies have been conducted to investigate whether miRNA therapy can serve as curative measures. Mimics of miR-150 and miR-29c-3p, both of which are TS-miRNAs in various cancers [112,126,127], have been observed to reduce inflammatory cytokines and microglial inflammasome activation, respectively [128,129]. Hu et al. found that the administration of mimics of miR-425, a TS-miRNA that is downregulated in cancer [130], intracerebrally improved locomotive behaviors and reduced DA neurodegeneration and necroptosis [131].

The dysregulation of miRNA is shown to be associated with ALS, also known as Lou Gehrig’s Disease, which is a progressive and fatal disease that affects the neurons in the brain and spinal cord. The motor neuronal damage leads to weakness, paralysis, and eventually death within 5 years of symptom onset. Some studies have found a positive correlation between the expression of miR-338-3p and miR-143-3p in CSF and serum from ALS patients [132]. As illustrated in many studies, miR-338, miR-142, miR-183, and let-7d play a role in neurodegeneration and apoptosis, while miR-206, miR-133a, miR-133b, and miR-27a are specifically expressed in striated muscle proliferation and at the muscle level [132]. The upregulation of miR-206, miR-133a, miR-133b, and miR-27a and inflammatory miRNAs (miR-155, miR-146a, and miR-221) were discovered in ALS patients with an earlier age of onset and longer disease duration [132]. In the peripheral tissues of ALS patients, eight miRNAs (miR-338-3p, miR-451, miR-1275, miR-328, miR-638, miR-149, miR-665, and miR-583) were deregulated in ALS patients. Among these, miR-338-3p was found in the brain tissues of ALS patients [133].

The dysregulation of miRNA is also shown to be associated with HD, an intractable neurodegenerative disease caused by CAG repeat expansion in the huntingtin gene located on chromosome 4p16.3. Patients present with cognitive defects and motor control impairment due to the progressive loss of cortical and striatal neurons [134]. The huntingtin protein was demonstrated to bind with repressor element 1 silencing transcription factor (REST) in neurons. In control individuals, the huntingtin protein sequesters REST in the cytoplasm and prevents the repressor from binding to DNA. In contrast, HD patients have mutant huntingtin protein that does not inhibit REST, which then permits REST to relocate to the nucleus of HD neurons and downregulate many of its target genes. One of them is BDNF, which is critical for the survivability of neurons [135]. Johnson et al. were able to identify a set of neuron-specific REST-target miRNAs in the human genome: miR-9-1, 9-3, 29a, 29b-1, 124a-1, 124a-2, 124a-3, 132, 135b, 139, 203, 204, 212, 330, and 346. Among these, miR-29a, miR-124a, miR-132, and miR-330 were found to be decreased in animal models for HD; however, only the downregulation of miR-132 was confirmed in human samples [136]. The expression profile of miRNAs in the frontal cortex and striatum of HD patients was also analyzed using qRT-PCR, RNA sequencing, and microarray. In all three of these different techniques, miR-100, miR-151-3p, miR-16, miR-219-2-3p, miR27b, miR-451, and miR-92a were found to be overexpressed in HD brain tissue [137]. In contrast, miR-128, miR-139-3p, miR-222, miR-382, miR-433, and miR-483-3p levels were decreased in the brain [137]. Other studies also found that miR-22, miR-29c, miR-132, miR-138, miR-218, miR-344, and miR-674 are downregulated in rodent models with HD [138,139]. Studies targeting specific miRNAs in HD models, such as miR-132, a TS-miRNA decreased in various types of malignancies [140,141], have shown HD symptom relief and stunting of HD progression [139].

## 4. MiRNAs Consistently Upregulated or Downregulated in Both Cancers and Neurological Disorders

The concept of ACCD suggests that cancers and neurological disorders share a common mechanism of dysregulated cell cycles, leading to tumorigenesis and progression in cancers and neuronal cell death in neurological disorders. With kinase/oncoproteins and TS being an integral part of the cell cycle regulation, we hypothesize that there will be a common set of oncogenic miRNAs that are upregulated and a common set of tumor suppressive miRNAs that are downregulated in both cancers and neurological disorders. We then identify the overlap in upregulated miRNAs and downregulated miRNAs in cancers and neurological disorders. A total of 273 miRNAs were found to be consistently upregulated in cancers only, while a total of 239 miRNAs were found to be consistently upregulated in neurological disorders only. The following 31 miRNAs were consistently upregulated in both cancers and neurological disorders across all disease types, studies, and sample types: miR-18a-5p, miR-18b-5p, miR-19a-3p, miR-20a-5p, miR-22-5p, miR-105-5p, miR-126-5p, miR-181a-5p, miR-223-5p, miR-323b-3p, miR-372-3p, miR-373, miR-455-3p, miR-488, miR-549, miR-582-5p, miR-585, miR-595, miR-627-5p, miR-671-5p, miR-762, miR-877-5p, miR-941, miR-1225-5p, miR-1249, miR-3613-5p, miR-4317, miR-4435, miR-iR-4293, let-7e-5p, and mIR-17-92 cluster (Table 1a). By contrast, a total of 269 miRNAs were found to be consistently downregulated in cancers only, while a total of 182 miRNAs were found to be consistently downregulated in neurological disorders only. The following 17 miRNAs were consistently downregulated in both cancers and neurological disorders across all disease types, studies, and sample types: miR-28-5p, miR-148-3p, miR-199b-3p, miR-208b, miR-219-5p, miR-320b, miR-323a-3p, miR-330, miR-374b, miR-449b-5p, miR-493-5p, miR-509-3p, miR-511, miR-575, miR-1297, miR-4487, and let-7i-5p (Table 1b). Since the downregulated miRs typically function as tumor suppressors, elevating those TS-miRNAs has the potential to treat both cancers and neurological disorders.

In general, upregulated miRNAs in cancers may be responsible for the promotion of oncomiRs, which results in unlimited tumor cell division in cancers [142], whereas downregulated miRNAs in cancers often serve as TS, and loss of TS-miRNAs play critical roles in uncontrolled tumor growth [143]. It was reported that miR-15/16 triggered apoptosis via inhibiting oncogenes, such as cyclin D1, CDC2, JUN, ETS1, MCL1, and others [142]. MiR-15/16 also repressed the expression of ROR1, a receptor for Wnt5a that promotes cell proliferation in leukemia and anoncoembryonic protein expressed in chronic lymphocytic leukemia (CLL) cells, as miR-15/16 expression is inversely related to ROR1 levels [142]. It was also reported that miR-140 targeted BCL9 and BCL2 and prevented colorectal cancer progression, miR-148a targeted BCL2 in non-small cell lung cancer, and miR-340 decreased BCL2 and increased Bax to induce cell apoptosis in ovarian cancer [144].

TS-miRNAs can block cell proliferation by blocking the WNT/B-catenin pathway in cancers. In particular, miR-200 targeted B-catenin, and miR-19 inhibited myocyte enhancer factor 2D (MEF2D) to block the Wnt pathway, while miR-133a-5p targeted TCF (which enhanced the transcription of oncogenes), thereby stopping cell proliferation in gastric cancer [145]. TS-miRNAs can also inhibit the endothelial-to-mesenchymal transition (EMT), which is critical for cancer metastasis. The miR-200 family inhibits EMT by directing decreasing amounts of zinc-finger E-box-binding homeobox1 (ZEB1) and SIP [146]. ZEB1 itself is capable of providing negative feedback to regulate the expression of miR-200. Thus, the disruption of ZEB1 has been shown to prevent EMT. Another factor that is critical for EMT maintenance is TBF-B, which downregulates miR-200 via the reversible DNA methylation of miRNA-200 [147].

To our knowledge, no other studies have explicitly linked the function of miRNAs as TSs to neurological disorders, except for our patent that demonstrated that elevating TS-miR-125b improves outcomes after TBI in rats by downregulating multiple oncogenic kinases (e.g., Mknk2, SFK, others) [62]. Instead, most studies have linked functions related to TS-miRNAs, such as apoptosis, to neurological disorders. For example, one study reported that miR-411 attenuated inflammatory damage and apoptosis in spinal cord injury in animal models [148]. Other studies showed that miR-124 may trigger anti-inflammatory responses and attenuate apoptosis in AIS [66].

## 5. Clinical Trials of miR drugs for Neurological Disorders

Although a large number of experimental studies demonstrated the robust efficacy of miRNA drugs for the treatment of neurological disorders, only three miRNA drugs have been tested so far in clinical trials for neurological indications (Table 2). One of the latest ongoing U.S. clinical trials to prevent HD progression involves AMT-130, which is a gene therapy that utilizes adeno-associated viral vector serotype 5 (AAV5) to deliver artificial miRNA (hsa-pre-miR-451a scaffold) that targets HTT to ultimately lower HTT [149] (NCT04120493) and a similar clinical trial is ongoing in Europe (NCT05243017). Of note, miR-451a is a known TS-miRNA in multiple cancers [150,151,152]. Though the trial is not yet complete, this approach has demonstrated success in reducing HTT expression in other models [153,154]. There are a few other clinical trials examining the use of miR therapies in other neurological disorders, such as ALS and epilepsy. One phase 1/2 clinical trial starting in 2024 (NCT06100276) and projected to end in 2027 is examining the safety, tolerability, and efficacy of AMT-162, a gene therapy that utilizes adeno-associated viral vector serotype 5 (AAV9) to deliver miRNA, which silences the SOD1 gene, in patients with ALS with the SOD1 mutation. One phase 1/2b clinical trial that started in 2023 and is projected to end in 2027 (NCT06063850) is examining the safety, tolerability, and efficacy of AMT-260, a gene therapy utilizing AAV9 to deliver miRNA to silence the GRIK2 gene, in patients with unilateral refractory mesial temporal lobe epilepsy.

There are a few major challenges that prevent the clinical development of miRNA therapeutics. First, the development of effective and safe delivery methods of miRNAs presents a challenge [155]. It is crucial to design tissue-specific targeting, as this will reduce off-target effects and decrease toxicity while improving efficacy [155]. Since oligonucleotides tend to be hydrophilic and negatively charged, another challenge that arises includes crossing the cell membrane to reach its target binding sites [156]. Ways of improving the ability of miRNAs to cross the cell membrane include using lipid nanoparticles and polymer-based carriers and the modification of the miRNA [156]. Secondly, it may be difficult to identify the most ideal miRNAs to target [155]. This is due to the heterogeneity of miRNA expression since multiple conditions, such as hypoxia and inflammation, can influence miRNA expression [155]. However, new next-generation RNA sequencing may help mitigate this issue [155]. Moreover, several databases and computational pipelines are being used to design synthetic miRNAs. For example, Laganà et al. present miR-Synth, a computational resource for the design of synthetic miRNAs able to target multiple genes in multiple sites. The proposed strategy constitutes a valid alternative to the use of siRNA, allowing the employment of a single molecule for the inhibition of multiple to numerous targets. This may represent a great advantage in designing therapies for diseases caused by crucial cellular pathways altered by numerous dysregulated genes [157]. Understanding these mechanisms will allow us to develop targeted solutions at the transcriptional level and make breakthroughs in therapeutics.

## 6. Conclusions

In summary, miRNA therapeutics is at the infancy stage in drug development, with some TS-miRNAs, which were originally identified in cancers, being investigated in clinical and preclinical trials for neurological treatments. As an emerging therapeutic approach, TS-miRNAs have the potential to target multiple oncogenic kinases at a time, which makes them a promising candidate to rescue neurons for the treatment of neurological diseases that typically involve multiple harmful pathways of brain injury. The understanding of miRNA characterization and fundamental scientific knowledge have grown explosively over the past few years. As such, the potential for miRNAs in the application of personalized medicine is being recognized due to their involvement in the regulation of genes, ease of access as a biomarker, and ability to influence multiple targets as therapeutics. With the advent of new techniques, being able to overcome the barriers of delivery, specific targeting, and identifying ideal miRNA candidates will immensely help promising preclinical miRNA drugs (including TS-miRNAs for neurological treatment) reach clinical trials and eventually FDA approval.

## Figures and Tables

**Table 1:** (a) miRNAs upregulated in cancers and neurological disorders; (b) miRNAs downregulated in cancers and neurological disorders.

**(a)**		
**miRNAs Upregulated in Cancers Only**	**miRNAs Upregulated in Both Cancers and Neurological Disorders**	**miRNAs Upregulated in Neurological Disorders Only**
miR-BART7, miR-BART13, miR-2, miR-3, miR-3P, miR-9-1, miR-9-2, miR-9-3, miR-15b-5p, miR-16-1-3p, miR-16-2, miR-16-2-3p, miR-18, miR-19b, miR-19b-3p, miR-20, miR-20a-3p, miR-20b-3p, miR-21-3p, miR-24-2, miR-24-3p, miR-25a, miR-25-5p, miR-27, miR-27a, miR-27-3p, miR-28, miR-28-3p, miR-29a-3p, miR-29c-3p, miR-30b, miR-30c-2, miR-30e-3p, miR-31-5p, miR-32-5p, miR-33, miR-33b-3p, miR-45, miR-48, miR-59, miR-81a, miR-92a-3p, miR-92b, miR-93-5p, miR-95-3p, miR-96-3p, miR-96-5p, miR-98-5p, miR-106a-3633, miR-106b-25, miR-125b-1-3p, miR-126a, miR-129-1, miR-129-2, miR-130, miR-130a-3p, miR-131a, miR-132-5p, miR-135, miR-135a, miR-135b, miR-138-1-3p, miR-138-2-3p, miR-138b, miR-141b, miR-145b, miR-146-5p, miR-146b-3p, miR-146b-5p, miR-148b, miR-151, miR-151-3p, miR-151a, miR-151a-3p, miR-152-3p, miR-159, miR-159a, miR-181a, miR-181a-2-3p, miR-181b-5p, miR-185-5p, miR-187-5p, miR-189-5p, miR-190, miR-194-5p, miR-196, miR-196b, miR-197-5p, miR-199-5p, miR-199-s, miR-203a, miR-203b, miR-208a, miR-208a-3p, miR-210, miR-210-3p, miR-215-5p, miR-218-1, miR-221/222 cluster, miR-222-3p, miR-222-5p, miR-223-3p, miR-244, miR-275, miR-300, miR-301, miR-301b, miR-301b-3p, miR-302-367 cluster, miR-302-3p, miR-302b-3p, miR-320e, miR-325, miR-339-5p, miR-361, miR-365a, miR-365b, miR-367, miR-369-3p, miR-370-3p, miR-371, miR-371a-3p, miR-371a-5p, miR-373-3p, miR-373-5p, miR-374a, miR-378a-5p, miR-378d, miR-409, miR-411, miR-412, miR-412-3p, miR-421, miR-423-3p, miR-423-5p, miR-449, miR-450a, miR-450b-5p, miR-452-5p, miR-466, miR-483, miR-483-5p, miR-485-3p, miR-485-5, miR-493-3p, miR-498, miR-500, miR-500a, miR-502-3p, miR-503-5p, miR-505-3p, miR-506-514 cluster, miR-512-5p, miR-513-1, miR-513-2, miR-514, miR-516a-3p, miR-517, miR-517b-3p, miR-518a, miR-518c, miR-519a, miR-520c-3p, miR-520f, miR-520g, miR-520h, miR-548a, miR-548ah, miR-548ar, miR-548b, miR-551b-3p, miR-551b-5p, miR-556-5p, miR-574-5p, miR-582-3p, miR-584-5p, miR-589, miR-592, miR-615-3p, miR-616, miR-618, miR-626, miR-628, miR-628-5p, miR-629-5p, miR-633, miR-635, miR-640, miR-642a-5p, miR-642b-3p, miR-645, miR-647, miR-657, miR-6741-3p, miR-675-3p, miR-711, miR-764, miR-767, miR-769, miR-857, miR-877-3p, miR-886-3p, miR-886-5p, miR-933, miR-938, miR-939, miR-1185-2-3p, miR-1234, miR-1247, miR-1248, miR-1254, miR-1269, miR-1273g-3p, miR-1275, miR-1293, miR-1303, miR-1307, miR-1323, miR-1537, miR-1537-3p, miR-1825, miR-1908, miR-1908-5p, miR-3065, miR-3131, miR-3136, miR-3141, miR-3144-3p, miR-3147, miR-3151, miR-3153, miR-3162, miR-3176, miR-3177-3p, miR-3189, miR-3200-5p, miR-3201, miR-3613-3p, miR-3917, miR-3976, miR-4267, miR-4270, miR-4283, miR-4289, miR-4418, miR-4429, miR-4465, miR-4484, miR-4644, miR-4652, miR-4664-3p, miR-4665-5p, miR-4709, miR-4764-3p, miR-5001-5p, miR-5100, miR-5191-3p, miR-5694, miR-6796-3p, miR-6826, miR-6852, miR-6875, miR-7641, miR-7702, let-5p, let-7b, let-7b-3p, let-7c-5p, let-7d-5p, let-7f-2, let-7f-5p, let-7g	miR-18a-5p, miR-18b-5p, miR-19a-3p, miR-20a-5p, miR-22-5p, miR-105-5p, miR-126-5p, miR-181a-5p, miR-223-5p, miR-323b-3p, miR-372-3p, miR-373, miR-455-3p, miR-488, miR-549, miR-582-5p, miR-585, miR-595, miR-627-5p, miR-671-5p, miR-762, miR-877-5p, miR-941, miR-1225-5p, miR-1249, miR-3613-5p, miR-4317, miR-4435, miR-iR-4293, let-7e-5p, mIR-17-92 cluster	miR-3p-57664, miR-7-1-3p, miR-7b-5p, miR-9-3p, miR-9a-3p, miR-10, miR-10a, miR-15a, miR-17-5p, miR-18b, miR-21a-5p, miR-25, miR-26a-2, miR-26b-3p, miR-27a-5p, miR-27b, miR-27b-5p, miR-30a-3p, miR-30a-5p, miR-30c-2-3p, miR-30d, miR-30e-5p, miR-32, miR-32-3p, miR-93a-5p, miR-99a, miR-99a-5p, miR-99b, miR-99b-5p, miR-101-3p, miR-101b-3p, miR-106-5p, miR-124a, miR-124-2, miR-124-3, miR-125a-5p, miR-125b-2, miR-128b, miR-130b, miR-133, miR-133a-1, miR-133a-2-3p, miR-134-5, miR-135b-3p, miR-136, miR-136-5p, miR-139, miR-142, miR-142a-5p, miR-145, miR-146b, miR-150, miR-152, miR-154-5p, miR-155-5p, miR-181a-3p, miR-181b-1-3p, miR-181c-3p, miR-184, miR-186-3p, miR-187, miR-191, miR-192-3p, miR-192-5p, miR-193b-5p, miR-194, miR-194-3p, miR-195, miR-195-3p, miR-196a, miR-199a, miR-199b, miR-200b, miR-200c, miR-203, miR-203-3p, miR-204-5p, miR-206, miR-208a-5p, miR-220c, miR-290, miR-211, miR-211-5p, miR-219a.2-3p, miR-219a-5p, miR-292-5p, miR-298, miR-300-3p, miR-302d-3p, miR-322, miR-323a-5p, miR-328-5p, miR-330-3p, miR-339, miR-339-3p, miR-340-3p, miR-345-3p, miR-362, miR-362-3p, miR-363-3p, miR-365a, miR-375, miR-376b-3p, miR-378c, miR-381, miR-382-3p, miR-424, miR-424-5p, miR-425, miR-433-3p, miR-434-3p, miR-451-5p, miR-454-3p, miR-455-5p, miR-486, miR-488-3p, miR-490-3p, miR-494, miR-495-3p, miR-499, miR-499a-3p, miR-499a-5p, miR-500a-3p, miR-505-5p, miR-509-3-5p, miR-513a-5p, miR-518f-3p, miR-519, miR-520d-3p, miR-520f-3p, miR-525-5p, miR-532, miR-532-3p, miR-545, miR-548at-5p, miR-548d, miR-550, miR-552, miR-553, miR-572, miR-579, miR-579-3p, miR-590-5p, miR-601, miR-602, miR-603, miR-611, miR-613, miR-615-5p, miR-617, miR-623, miR-627, miR-628-3p, miR-629-3p, miR-637, miR-638, miR-641, miR-656-3p, miR-659, miR-660, miR-660-5p, miR-663a, miR-665, miR-668, miR-671, miR-671-3p, miR-675, miR-682, miR-686, miR-762, miR-765, miR-874, miR-883a-3p, miR-883b-3p, miR-891, miR-920, miR-923 miR-942, miR-943, miR-1183, miR-1184, miR-1185-1-3p, miR-1202, miR-1224, miR-1246, miR-1249, miR-1255b, miR-1261, miR-1274a, miR-1274b, miR-1277-3p, miR-1285, miR-1285-5p, miR-1289, miR-1290, miR-1291, miR-1321, miR-1843a-5p, miR-2861, miR-3190-3p, miR-3195, miR-3196, miR-3615, miR-3646, miR-3928-5p, miR-3939, miR-4306, miR-4433b-3p, miR-4449, miR-4521, miR-4649-5p, miR-4669, miR-4674, miR-4747-3p, miR-5001-3p, miR-5010-3p, miR-6096-5p, miR-6716-3p, miR-6736-3p, miR-6740-3p, miR-6747-3p, miR-6750-5p, miR-6753-3p, miR-6754-3p, miR-6761-3p, miR-6762-3p, miR-6777-3p, miR-6778-3p, miR-6787-3p, miR-6836-3p, miR-6867-5p, miR-6875-3p, miR-8082, miR-B19527a, let-7-c-3p let-7e, let-7g-3p, miR-PC-5p-12969, miR-23-27-24 cluster
**(b)**		
**miRNAs Downregulated in Cancers Only**	**miRNAs Downregulated in Both Cancers and Neurological Disorders**	**miRNAs Downregulated in Neurological Disorders Only**
miR-1-3p, miR-6-3p, miR-10, miR-15-5p, miR-15/16 cluster, miR-15a-3p, miR-16-1, miR-16.1, miR-23a-3p, miR-24-1-5p, miR-26, miR-26a-5p, miR-27a-5p, miR-29b, miR-29b-1-5p, miR-29c-5p, miR-30, miR-30b-3p, miR-30b-5p, miR-30c-2-3p, miR-33a-5p, miR-34b-3p, miR-34b-5p, miR-92, miR-92a cluster, miR-93-3p, miR-99-3b, miR-99b-5p, miR-103b, miR-104-5p, miR-122a, miR-124-5p, miR-126b, miR-127-3p, miR-128-1, miR-129-3p, miR-129b-5p, miR-130b-3p, miR-130b-5p, miR-132-5p, miR-133, miR-133a, miR-133a-3p, miR-134-5p, miR-135-5p, miR-136, miR-136-5p, miR-137, miR-138-5p, miR-145, miR-147, miR-147b, miR-148a-3p, miR-149-5p, miR-152, miR-153, miR-153-3p, miR-181a-3p, miR-181d-5p, miR-188, miR-193-3p, miR-193-5p, miR-193a, miR-196b-5p, miR-199-3p, miR-216-5p, miR-216a, miR-216a-3p, miR-216b, miR-217, miR-218-5p, miR-219a-2-3p, miR-220, miR-220a, miR-220b, miR-269-3p, miR-296-3p, miR-299, miR-302, miR-302a, miR-302b, miR-302c, miR-320a-3p, miR-323, miR-323-3p, miR-323-5p, miR-328, miR-328-3p, miR-330-3p, miR-330-3p-3p, miR-330-5p, miR-333-3p, miR-335-5p, miR-336, miR-337, miR-339-3p, miR-342, miR-362, miR-363, miR-365b-3p, miR-365b-5p, miR-374c, miR-374c-5p, miR-376, miR-376a-3p, miR-381-3p, miR-382-5p, miR-383-5p, miR-384, miR-385, miR-397a, miR-397b, miR-397c, miR-411-5p, miR-424-3p, miR-429-5p, miR-432, miR-433-3p, miR-448, miR-449b, miR-449c, miR-451, miR-451a.1, miR-454-3p, miR-485-5p, miR-486-3p, miR-487b, miR-488-3p, miR-488-5p, miR-489, miR-489-3p, miR-490-3p, miR-491, miR-491-3p, miR-491-5p, miR-497-5p, miR-506-3p, miR-508-3p, miR-509-5p, miR-509-3-5p, miR-511-5p, miR-513, miR-513c-5p, miR-514a-3p, miR-516b, miR-519, miR-520a-3p, miR-524-5p, miR-526b, miR-526b-5p, miR-532-5p, miR-539, miR-544, miR-545, miR-548b-5p, miR-548c-3p, miR-551b, miR-564, miR-573, miR-574, miR-579-3p, miR-582, miR-584, miR-590-3p, miR-593-3p, miR-596, miR-599, miR-601, miR-605, miR-610, miR-612, miR-613, miR-622, miR-625, miR-625-5p, miR-627, miR-628-3p, miR-632, miR-634, miR-636, miR-637, miR-639, miR-650, miR-652-5p, miR-654-5p, miR-655, miR-656, miR-661, miR-665, miR-708, miR-708-5p, miR-744, miR-744-5p, miR-760, miR-768-3p, miR-770-5p, miR-802, miR-874, miR-877, miR-886-3p, miR-887, miR-891a, miR-892b, miR-936, miR-937, miR-937-5p, miR-942, miR-1179, miR-1180-3p, miR-1182, miR-1203, miR-1205, miR-1226, miR-1227, miR-1228-5p, miR-1231, miR-1250, miR-1257, miR-1260, miR-1266, miR-1271, miR-1274a, miR-1280, miR-1283, miR-1287, miR-1296, miR-1301-3p, miR-1302, miR-1321, miR-1587, miR-1913, miR-2682-3p, miR-2861, miR-3065-5p, miR-3156-3p, miR-3170, miR-3185, miR-3196, miR-3612, miR-3614, miR-3651, miR-3662, miR-3666, miR-3714, miR-3928, miR-3960, miR-4259, miR-4282, miR-4324, miR-4430, miR-4458, miR-4467, miR-4478, miR-4485-5p, miR-4488, miR-4501, miR-4633-5p, miR-4635, miR-4647, miR-4706, miR-4730, miR-4731, miR-5096, miR-6086, miR-6503, miR-6510, miR-6511b-5p, miR-6887-5p, let-7a	miR-28-5p, miR-148-3p, miR-199b-3p, miR-208b, miR-219-5p, miR-320b, miR-323a-3p, miR-330, miR-374b, miR-449b-5p, miR-493-5p, miR-509-3p, miR-511, miR-575, miR-1297, miR-4487, let-7i-5p	miR-2-5p, miR-7, miR-7c-5p, miR-7g, miR-9a-5p, miR-15, miR-15b, miR-15b-3p, miR-15b-5p, miR-16-2-3p, miR-16-5p, miR-17-3p, miR-18a, miR-19b-5p, miR-22-5p, miR-23b, miR-23b-3p, miR-26a, miR-26a-1, miR-26a-2-5p, miR-26b-5p, miR-27a-3p, miR-29, miR-30c, miR-31-5p, miR-33b-5p, miR-92a-5p, miR-92b, miR-96, miR-96-5p, miR-98, miR-98-5p, miR-101, miR-103, miR-106a-5, miR-106b-3p, miR-112, miR-122, miR-125, miR-125a, miR-125a-3p, miR-125b-1-3p, miR-126-3p, miR-128-2-5p, miR-130a, miR-130-b-5p, miR-134-3p, miR-139-3p, miR-140, miR-146-5p, miR-146a-3p, miR-146b-5p, miR-148b, miR-148b-3p, miR-148b-5p, miR-149, miR-150-3p, miR-151-3p, miR-151b, miR-161, miR-181-3p, miR-181-5p, miR-182, miR-182-5p, miR-183, miR-183-5p, miR-186, miR-186-5p, miR-187-3p, miR-187-5p, miR-190, miR-192, miR-193a-5p, miR-193b, miR-199a-5p, miR-203a-3p, miR-203b-5p, miR-204, miR-208a, miR-208b-3p, miR-212, miR-212-3p, miR-219, miR-221-5p, miR-298-5p, miR-299-5p, miR-301, miR-320, miR-320a, miR-320c, miR-320e, miR-325-5p, miR-331-3p, miR-335-3p, miR-337-5p, miR-340, miR-340-5p, miR-342-3p, miR-351-3p, miR-352, miR-362-5p, miR-370-3p, miR-374a, miR-374b-5p, miR-379, miR-380, miR-423-3p, miR-423-5p, miR-425-p, miR-433, miR-483-3p, miR-485, miR-486-1, miR-487a, miR-487b-3p, miR-493, miR-500, miR-501-5p, miR-502, miR-502-3p, miR-502-5p, miR-519e, miR-543, miR-545-3p, miR-574-3p, miR-576-5p, miR-598, miR-598-3p, miR-605-5p, miR-626, miR-629, miR-652, miR-669c-5p, miR-744, miR-744-3p, miR-764-3p, miR-767, miR-769, miR-769-5p, miR-873-3p, miR-877-3p, miR-885-3p, miR-885-5p, miR-886-5p, miR-1185-2-3p, miR-1224-5p, miR-1233-5p, miR-1234-3p, miR-1237-5p, miR-1249-3p, miR-1259, miR-1294, miR-1299, miR-1303-3p, miR-1306-3p, miR-1825, miR-1909-3p, miR-1915-3p, miR-3552, miR-3613-3p, miR-3916, miR-3935, miR-4299, miR-4322, miR-4422, miR-4429, miR-4448, miR-4530, miR-4745-5p, miR-4772-3p, miR-6131, miR-6236, miR-6805-5p, miR-6781-5p, miR-7046-3p, miR-7660-3p, miR-7665-5p, miR-7674-5p, miR-8071, let-7a-5p, let-7c, let-7g

**Table 2:** Study details of clinical trials on miRNA therapeutics in neurological disorders.

Clinical Trial NCT Number	Aims of Clinical Trial	Phase	Start and End Date	Status	Results
NCT04120493	Establish safety and proof-of-concept of AMT-130 in patients with early-stage HD.	Interventional (Phase I/II)	6 September 2019–June 2019 (estimated)	Recruiting	None
NCT05243017	Examine safety and efficacy of AMT-130 in patients with HD.	Interventional (Phase I/II)	7 October 2021–7 October 2027 (estimated)	Recruiting	None
NCT06100276	Examine safety, tolerability, and efficacy of AMT-162 in patients with ALS.	Interventional (phase 1/II)	1 Febuary 2024–27 Febuary 2032 (estimated)	Not Recruiting Yet	None
NCT06063850	Examine safety, tolerability, and efficacy of AMT-260 in patients with unilateral refractory mesial temporal lobe epilepsy.	Interventional (phase I/II)	17 November 2023–30 November 2026 (estimated)	Recruiting	None

HD = Huntington’s Disease, ALS = amyotrophic lateral sclerosis.

## Data Availability

Data sharing is not applicable.

## Supplementary Materials

The following supporting information can be downloaded at: https://www.mdpi.com/article/10.3390/ph17040426/s1, Table S1: MicroRNA fingerprints in different types of samples for each sub-type of cancers and neurological disorders.
